# Survival analysis of early intention of antenatal care among women in Bangladesh

**DOI:** 10.1038/s41598-024-55443-5

**Published:** 2024-02-27

**Authors:** Md. Ismail Hossain, Tanjima Rahman, Tahsin Shams Sadia, Ahmed Abdus Saleh Saleheen, Shuvongkar Sarkar, Maruf Khan, Tahsina Fariha Ohi, Iqramul Haq

**Affiliations:** 1 Department of Mathematics and Natural Sciences, BRAC University, Dhaka, 1212 Bangladesh; 2 Department of Statistics, Jagannath University, Dhaka, 1100 Bangladesh; 3 Department of Soil Science, University of Chittagong, Chattogram, 4331 Bangladesh; 4 Department of Agricultural Economics, Sher-e-Bangla Agricultural University, Dhaka, 1207 Bangladesh; 5 Department of Agricultural Statistics, Sher-e-Bangla Agricultural University, Dhaka, 1207 Bangladesh

**Keywords:** Early intention, Antenatal Care (ANC), Cox proportional hazard model, Maternal and child health, Health care, Public health

## Abstract

This study focuses on the importance of early and regular Antenatal Care (ANC) visits in reducing maternal and child mortality rates in Bangladesh, a country where such health indicators are a concern. The research utilized data from the Bangladesh Demographic and Health Survey (BDHS) conducted in 2017–18 and employed the Cox proportional hazard model to identify factors influencing women’s intention of ANC services. The results revealed that 40.4% of women engaged in at least one ANC activity during the first trimester, which, although higher than in other countries, falls below the global average. Notably, women between the aged of 25 and 29 years took 15% less time for their first ANC visit compared to their younger counterparts, suggesting higher awareness and preparedness in this age group. Education, both for women and their partners, had a significant influence on the intention to visit ANC early. Women in the poor wealth quantile exhibited lower odds of seeking timely ANC, whereas those with a planned pregnancy were more likely to do so. Moreover, access to mass media decreased the timing of ANC visits by 26% compared to women who were not exposed. Moreover, living in rural areas was linked to a 17% delay in the timing of the first ANC visit compared to urban areas. These findings underscore the importance of addressing these determinants to improve the timeliness and accessibility of ANC services, thereby enhancing maternal and child health outcomes in Bangladesh.

## Introduction

Antenatal care (ANC) plays a crucial role in promoting a healthy pregnancy, offering pregnant women regular checkups from trained medical professionals starting from conception until labor begins^1,2^. Despite global efforts to improve maternal and child health, perinatal death rates have surged^3^, particularly in sub-Saharan Africa and south Asia, with 85% of maternal deaths predicted to occur in these regions by 2020^4^. Southern Asia, including Bangladesh, bears a substantial burden, accounting for one-third of global maternal and child deaths^5^. Delayed or inadequate healthcare for pregnant women contributes to poor outcomes of maternal and prenatal health^6–8^. To reduce these poor maternal and child outcomes, early antenatal care within the first three months of gestation is vital for timely detection and management of pregnancy-related complications. However, in many low and middle-income countries, the rate of early antenatal care attendance remains low.

The prevalence of early antenatal care visits is approximately 43% globally^9^, whereas in sub-Saharan countries, it is only about two quarters of the total^1^. A recent study conducted in Papua New Guinea revealed the prevalence of early ANC intention is 23%^10^. According to studies done in Uganda, the proportion of early ANC visit was 36%^11^. Likewise, another study conducted in Northwest Ethiopia reported that 52% of women booked their first ANC visit after 4 months of pregnancy^12^. A recent study conducted in India, around one-quarter of pregnant women booked ANC services within the first trimester at the national level^13^. Over the past 14 years (2004–2017) in Bangladesh, the rate of adequate antenatal care visits has been increasing, but the percentage of early intention of ANC attendance remains relatively low^14^. According to the recent Bangladesh Demographic and Health Survey report, the median months pregnant at first visit is 5 months^14^. Indeed, like other Low- and Middle-Income Countries (LMICs), studying and addressing the issue of early antenatal care (ANC) intention is of paramount importance for Bangladesh. Improving early ANC attendance can have significant implications for maternal and prenatal health outcomes, and it remains a crucial area of research and intervention for enhancing maternal and child health in the country.

Building upon the significance of early antenatal care (ANC) attendance in Bangladesh, this study aims to assess the prevalence and identify the risk factors associated with early intention of ANC among mothers in the country. Through this research, we seek to contribute valuable insights to inform evidence-based interventions and policies that can enhance maternal and prenatal health outcomes and ultimately benefit the well-being of mothers and their children in Bangladesh. In the past, several researchers have tried to determine the actual risk factors for early intention of ANC by using various techniques and different statistical models. A popular binary logistic regression model was used in most of the studies^15,16^. In a recent study conducted among 512 mothers residing in northwest Ethiopia, the findings indicate that women with secondary education exhibit more than double the early intention for ANC compared to those with lower levels of education^17^. Notably, planned pregnancy was significantly correlated with an early intention for ANC^18^. In a hospital-based cross-sectional study in Myanmar, classical logistic regression revealed that residence, education of the pregnant woman, occupation of the husband, parity, and planned pregnancy were significant determinants of receiving early ANC^19^. However, the early intention of ANC can be classified as time to event data. By considering this survival time nature, in this study, we employed a Cox proportional hazard model to examine the time to early ANC intention among women in Bangladesh. This model allows us to account for the varying follow-up times of participants and provides valuable insights into the factors that influence the timing of ANC attendance. By employing the Cox proportional hazard model in our study, we aim to provide a more nuanced understanding of the risk factors influencing early ANC intention in Bangladesh. It is worth noting that previous research in other countries has predominantly used binary logistic regression models to explore factors influencing early ANC attendance^20,21^. However, in the context of Bangladesh, such comprehensive investigations into the determinants of early ANC intention have been limited. Thus, this study fills a critical knowledge gap by utilizing the more appropriate Cox proportional hazard model, which is particularly well-suited for analyzing time-to-event data and aligns with the cross-sectional nature of ANC intention.

## Materials and methods

### Data sources

The analysis of this study was done by using data from Bangladesh Demographic and Health Survey (BDHS), 2017–18, which was a large scale nationally representative cross-sectional study^14^. ICF implements the DHS program and provided technical assistance through the program. The United States Agency for International Development (USAID) is funding the survey.

### Sample design

**Figure 1:**
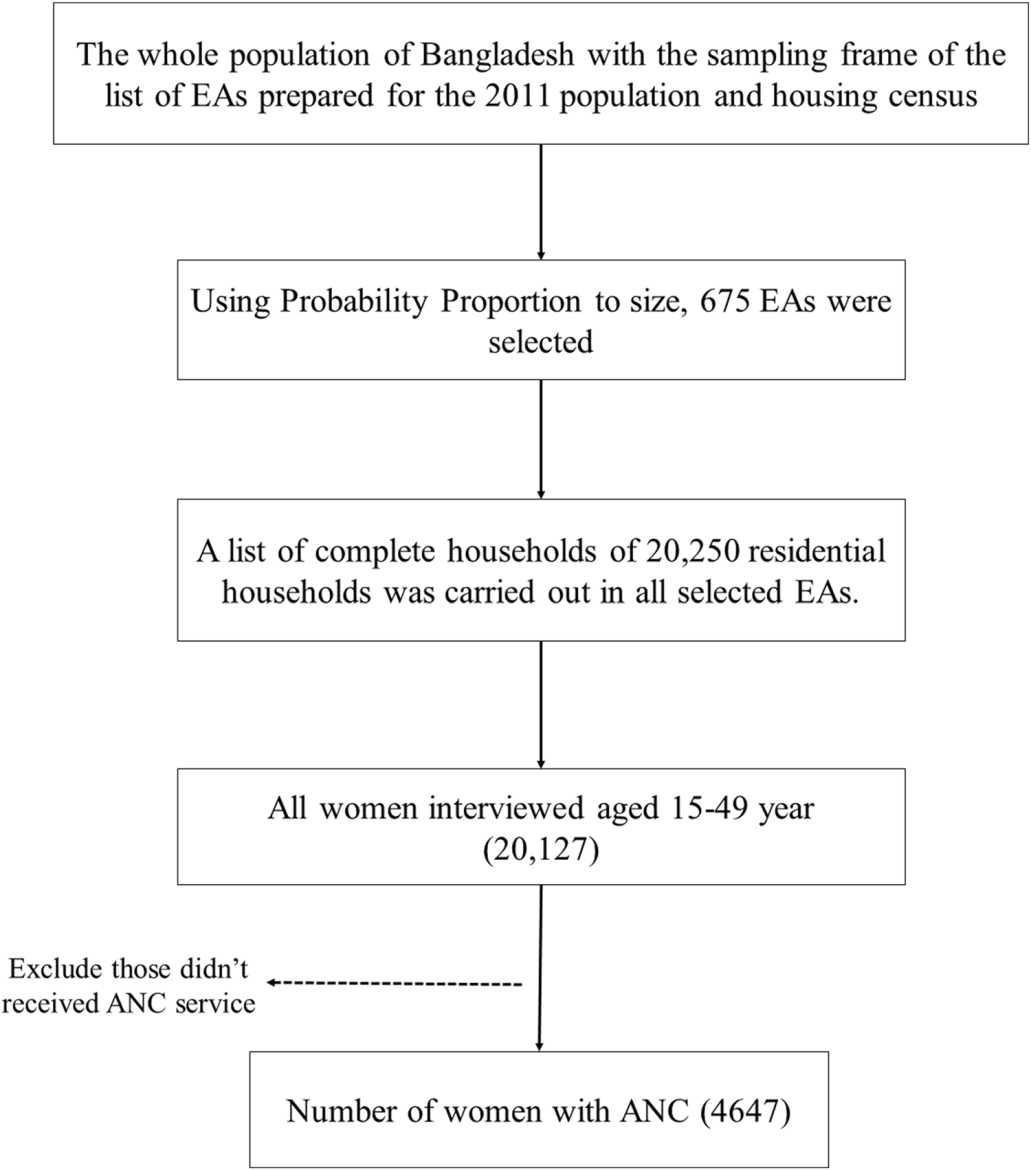
Study population and sample selection procedure for this study.

This cross-sectional survey was conducted in urban and rural areas of Bangladesh and used a two-stage stratified sampling design. In the first stage, 675 enumeration areas were selected, and in the second stage, 30 households were selected from each enumeration area. The survey was conducted in 20,250 households and 20,127 reproductive aged women were interviewed^14^. Due to the collection of samples from a finite population, the estimation procedure and testing of the data needed suitable sampling weight adjustment. The specific focus of this study involved a target sample comprising women of reproductive age who had received antenatal care and had given birth to live infants within the three years preceding the survey. According to this criterion, the study’s weighted sample size comprised 4,647 women. The exclusion and inclusion processes are depicted in Fig. 1.

### Dependent variable

This study focused on the early intention of antenatal care (ANC) as the response variable. Early intention was defined as attending the initial ANC visit at 3 months of pregnancy or earlier. The time-to-event concept integrated the months of pregnancy and the survival status (classified as early or late intention). The pregnancy months for each woman, categorized as either early or not, were recorded during the interviews. For coding purposes, a “1” was assigned if a woman reported attending ANC at 3 months of pregnancy or earlier, and a “0” was assigned if she either did not attend ANC or attended after 3 months. In this context, women attending ANC at or before three months was considered an event, while women either did not attend ANC or attended after three months was considered right censoring.

### Independent variables

In accordance with prior research, this study incorporated a range of independent variables, including women's age group in years (15–24, 25–29, 30–49)^22^, women’s education (No education, Primary education, Secondary education, Higher)^23^, partner’s education (No education, Primary education, Secondary education, Higher)^23^, wealth status (Poor, Middle Rich)^23^, pregnancy intention (Unintended, Intended)^24^, working status (Yes, No)^22^, decision-making on health care (Yes, No), access to mass media (Yes, No)^22^, residence (Urban, Rural)^23^, and region (Southern, Central, Eastern, Northern). In this context, the northern region includes Rajshahi and Rangpur divisions, the eastern region comprises Chattogram and Sylhet divisions, the central region comprises Dhaka and Mymensingh divisions, and the southern region comprises Barisal and Khulna divisions.

### Statistical analysis

In this study, the log-rank test was employed to examine any significant differences in the timing of ANC intention across various socio-economic and demographic characteristics. The null hypothesis used in this test is that there lies no difference between the groups in the probability of an event at any point of time. Here the event indicates time taken for first ANC visit. A significant log-rank test suggests that there is a statistically significant difference in survival between the groups being compared. Finally, the factors affecting early intention of ANC is determined using the Cox Proportional Hazard (PH).

A valuable approach for identifying potential covariate effects on the hazard function is the Cox proportional hazards (PH) model. This model is semi-parametric because the covariates enter the model linearly, but the baseline hazard can take any form. Let, for the $i{\text{th}}$ women, the model indicator is $S_{i}$ for,

$$S_{i} = \left\{ {\begin{array}{*{20}c} {1;{\text{when woman reported attending ANC at }}3{\text{ months or earlier }}} \\ {0;{\text{when woman did not attending ANC at }}3{\text{ months or earlier }}} \\ \end{array} } \right.$$

The Cox proportional hazards model has the following functional form:

1 $$h\left( {\frac{t}{{Z_{i} }}} \right) = h_{0} \left( t \right){\text{e}}^{{\left( {\beta_{1} Z_{i1} + \beta_{2} Z_{i2} + \ldots \ldots \ldots + \beta_{k} Z_{ik} } \right)}}$$

2 $$or, \;\;\;\;h\left( {\frac{t}{{Z_{i} }}} \right) = h_{0} \left( t \right) {\text{e}}^{{\beta^{T} Z_{i} }}$$

Here, for $i{\text{th}}$ women (first ANC visit) at time $t$, the vector of the covariates is $Z_{i}$ and $\beta = \left( {\beta_{1} ,\beta_{2} , \ldots \ldots , \beta_{k} } \right)^{T}$. represent unknown regression coefficient assuming equal for the women at time point $t$. Where $h_{0} \left( t \right)$ is called the baseline hazard.

The SPSS (Statistical Package for Social Science) version 25 and R-programming version 4.0.0 was used for data management, analysis.

### Ethical approval

For this study, a publicly accessible secondary dataset was utilized, which was obtained from the Demographic and Health Surveys (DHS) Program website (https://dhsprogram.com/data/). Since the dataset was already available and no primary data collection was involved, no additional ethics approval was required for the use of this dataset.

## Results

Table 1 displays the percentage distribution of women of reproductive age who received antenatal care and gave live birth in the three years preceding the survey in Bangladesh, categorized by various sociodemographic and demographic characteristics. Notably, 72.3% of these women resided in rural areas, contrasting with 27.7% in urban areas, and the central region housed the majority (34.3%) with a smaller representation from the southern region (14.9%). The majority of women (53.7%) fell within the 15–24 age group, and a significant proportion had completed their secondary education (50.4%). A notable 63.4% of these women were unemployed. Regarding their partner’s education, primary (32%) and secondary (34.8%) levels were most prevalent. In terms of wealth status, the majority of women were from rich households (approximately 42%). Notably, a significant percentage of women (83.7%) made decisions about their own healthcare, and a large proportion expressed intentions for pregnancy (79.8%). Furthermore, 57.7% had exposure to mass media.

**Table 1:** Background characteristics and percentage distribution of early ANC intention by survival determinants of sampled women who received ANC during pregnancy period.

Variables	Frequency (%)	Early intention of receiving ANC	
		Yes (40.4%)	No (59.6%)
Women’s age group (in years)			
15–24	2494 (53.7)	39.9	60.1
25–29	1204 (25.9)	42.3	57.7
30–49	949 (20.4)	39.2	60.8
Women’s education			
No education	234 (5.0)	26.9	73.1
Primary	1219 (26.2)	30.2	69.8
Secondary	2340 (50.4)	39.3	60.7
Higher	854 (18.4)	61.6	38.4
Partner’s education			
No education	632 (13.6)	28.0	72.0
Primary	1488 (32.0)	31.1	68.9
Secondary	1618 (34.8)	41.6	58.4
Higher	909 (19.6)	61.8	38.2
Wealth status			
Poor	1787 (38.5)	30.3	69.7
Middle	908 (19.5)	37.4	62.6
Rich	1952 (42.0)	50.9	49.1
Pregnancy intention			
Unintended	938 (20.2)	33.9	66.1
Intended	3709 (79.8)	42.0	58.0
Working status			
Yes	1699 (36.6)	35.0	65.0
No	2948 (63.4)	43.5	56.5
Decision making on health care			
No	755 (16.3)	36.6	63.4
Yes	3891 (83.7)	41.1	58.9
Mass media access			
Yes	2680 (57.7)	46.5	53.5
No	1967 (42.3)	32.0	68.0
Residence			
Rural	3362 (72.3)	36.7	63.3
Urban	1285 (27.7)	49.9	50.1
Region			
Southern	690 (14.9)	38.3	61.7
Central	1592 (34.3)	47.4	52.6
Eastern	1302 (28.0)	37.4	62.6
Northern	1063 (22.9)	34.8	65.2

Additionally, Table 1 gives a complete overview of the percentage distribution of women indicating their intention to receive ANC at or before three months of pregnancy. Based on the data presented in Table 1, it is observed that approximately 42.3% of women aged 25 to 29 sought their initial antenatal care (ANC) during the early stages of pregnancy. Nearly two-thirds of women (61.6%) with higher education expressed an intention to receive their first antenatal care (ANC) within the first twelve weeks of pregnancy. Results from Table 1 show that a higher percentage of women whose partner had a secondary education level (61.8%) expressed interest in having ANC at an early pregnancy stage. Just over half of women (50.9%) from rich families received antenatal care (ANC) within the first three months of pregnancy. In contrast, women from middle-class (37.4%) and poor (30.3%) households exhibited less interest in obtaining ANC during the early stages of pregnancy. Only 42% of women who independently consented to becoming pregnant exhibited an early intention to receive their first antenatal care (ANC). Working women had a lower percentage of early intention to receive antenatal care (ANC) at 35%, compared to non-working women (43.5%). Additionally, 41.1% of women who actively make decisions about their healthcare expressed an early intention to receive ANC. Women with access to mass media demonstrated a higher interest in having an early intention to receive antenatal care (ANC) compared to those without such access (46.5% vs. 32%). Urban women showed a higher early intention of receiving their first antenatal care (ANC) at 49.9%, in contrast to rural women, who had a lower percentage at 36.7%. Women from the central region exhibited a higher early intention of receiving their first antenatal care (ANC) at 47.4%, followed by those from the southern region (38.3%), eastern region (37.4%), and northern region (34.8%).

**Figure 2:**
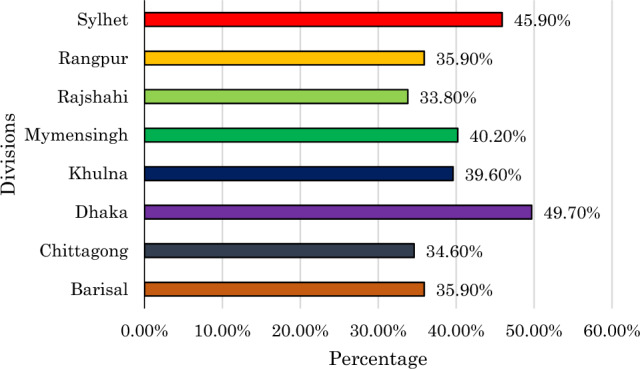
Percentage distribution of early ANC intention across division of Bangladesh.

Figure 2 displays regional variations in the early intention of Antenatal Care (ANC) visits among women in Bangladesh. The Dhaka division has the highest proportion, with 49.7%, followed by Sylhet (45.9%), Mymensingh (40.2%), and Khulna (39.6%). In contrast, Rajshahi (33.8%), Chittagong (34.6%), and Barisal (35.9%) have lower percentages, suggesting a need for targeted interventions to increase awareness and promote early ANC visits in these areas.

This study used the log-rank test to identify the significant difference between timing of ANC intention and various socio-economic and demographic characteristics, and the results are shown in Table 2. The results from Table 2 show that the intention of having early ANC significantly differed by all the factors, which are women’s age, women’s education, partner’s education, wealth status, pregnancy intention, working status, decision-making on health care, mass media access, residence, and region.

**Table 2:** Log-rank test for early ANC intention by covariates.

Variables	Median survival time	$\chi^{2}$ value (p-value)
Women’s age group (in years)		
15–24	5	7.6 (0.02)
25–29	4	
30–49	5	
Women’s education		
No education	5	288 (< 0.001)
Primary	5	
Secondary	4	
Higher	3	
Partner’s education		
No education	5	312 (< 0.001)
Primary	5	
Secondary	4	
Higher	3	
Wealth status		
Poor	5	224 (< 0.001)
Middle	5	
Rich	4	
Pregnancy intention		
Unintended	5	23.6 (< 0.001)
Intended	4	
Working status		
Yes	5	33 (< 0.001)
No	4	
Decision making on health care		
No	5	6.2 (0.01)
Yes	4	
Mass media access		
Yes	4	131 (< 0.001)
No	5	
Residence		
Rural	5	83.7 (< 0.001)
Urban	4	
Region		
Southern	4	23.4 (< 0.001)
Central	4	
Eastern	5	
Northern	5	

Table 2 demonstrates that the median pregnancy month of receiving the first ANC of women who had no education and a primary level of education, those whose partners were illiterate and had a primary level of education, and those who were from poor households were higher than other groups of the respective factors. Women with higher education had the smallest (3 months) median pregnancy month of receiving their first ANC compared with women with other levels of education. Whose partner had higher literacy had the least survival time, which was 3 pregnancy months, in contrast to other education levels. Women from 15 to 24 and 30 to 49 age groups had quite the same median pregnancy month of receiving their first ANC (5 months), while for women from 25 to 29 age groups it was slightly lower (4 months). The median pregnancy month of receiving their first ANC for women who had an intentional pregnancy is greater (4 months) than that of those who had it unintentionally (5 months). Those who worked outside had a larger survival time, i.e., 5 pregnancy months, than those who didn’t. All those women who didn’t have permission to make healthcare decisions had the highest median survival time compared to those who had it. Women with access to mass media had a comparatively lower pregnancy month of receiving their first ANC (4 months) than women who didn't have access to it. The median pregnancy month of receiving their first ANC for women living in urban areas is 4 months, which is smaller than for women who live in rural areas. Those who lived in the central region had the median pregnancy month of receiving their first ANC (4 months), while those who lived in the eastern and northern regions had the same number of months, i.e., 5 months.

This study employed the Cox proportional hazard model to examine the relationship between the early intention of seeking ANC and multiple factors that potentially influence this intention. The findings of the Cox proportional hazard model analysis, as presented in Table 3, highlight the specific factors that significantly impact the intention of early ANC.

**Table 3:** Cox’s proportional hazards model analysis: factors of early ANC intention in Bangladesh.

Variables	Coefficient	aHR (95% CI)	p-value
Women’s age group (in years)			
15–24 (Ref.)			
25–29	0.14	1.15 (1.03, 1.28)	0.01
30–49	0.08	1.09 (0.96, 1.23)	1.17
Women’s education			
No education	− 0.55	0.58 (0.43,0.76)	< 0.001
Primary	− 0.52	0.59 (0.50, 0.70)	< 0.001
Secondary	− 0.35	0.70 (0.62, 0.79)	< 0.001
Higher (Ref.)			
Partner’s education			
No education	− 0.51	0.60 (0.49, 0.73)	< 0.001
Primary	− 0.51	0.61 (0.52, 0.71)	< 0.001
Secondary	− 0.27	0.77 (0.68, 0.87)	< 0.001
Higher (Ref.)			
Wealth status			
Poor	− 0.19	0.83 (0.73, 0.95)	0.005
Middle	− 0.15	0.86 (0.76, 0.99)	0.02
Rich (Ref.)			
Pregnancy intention			
Unintended	− 0.24	0.78 (0.69, 0.88)	< 0.001
Intended (Ref.)			
Working status			
Yes	− 0.08	0.91 (0.82, 0.99)	0.04
No (Ref.)			
Decision making on health care			
No	− 0.12	0.88 (0.79, 1.03)	0.12
Yes (Ref.)			
Mass media access			
Yes	0.23	1.26 (1.13, 1.40)	< 0.001
No (Ref.)			
Residence			
Rural	− 0.19	0.83 (0.75, 0.92)	< 0.001
Urban (Ref.)			
Region			
Southern	0.02	1.02 (0.89, 1.18)	0.71
Central	0.22	1.26 (1.10, 1.43)	< 0.001
Eastern	0.11	1.11 (0.96, 1.27)	0.13
Northern (Ref.)			

aHR, adjusted hazard ratio; Ref, reference category.

The results in Table 3 demonstrate that women’s age significantly affects the time to intention of having an early ANC. For example, the study discovered that women aged 25–29 years took 15% (aHR = 1.15, 95% CI: 1.03, 1.28) less time for their initial ANC visit compared to those in the reference group (15–24 years). A significant association was found between the level of education and women’s time to first visit of ANC. Notably, women with secondary education exhibited a 30% longer time their initial ANC visit (aHR = 0.70, 95% CI: 0.62, 0.79) compared to those with higher education. Additionally, women whose partners had completed secondary education took 23% more time (aHR = 0.77, 95% CI: 0.68, 0.87) to receive antenatal care (ANC) compared to those whose partners had a higher level of education.

Regarding wealth status, women from poor and middle-income households took 17% (aHR = 0.83, 95% CI = 0.73, 0.95) and 14% (aHR = 0.86, 95% CI = 0.76, 0.99) higher time, respectively, of initiating their first ANC visit compared to women from rich households in Bangladesh. Additionally, the time taken for the first ANC visit was 22% higher (aHR = 0.78, 95% CI: 0.69, 0.88) for women who intentionally became pregnant compared to those with planned pregnancies. The time to early ANC visit was 9% higher for women who were employed compared to those who did not work outside the home. Women exposed to mass media were 26% (aHR = 1.26, 95% CI = 1.13, 1.40) more likely to receive early antenatal care (ANC) compared to those who were not exposed to mass media. Rural women in Bangladesh had a 17% higher time to their first ANC visit (aHR = 0.83, 95% CI: 0.75, 0.92) compared to their urban counterparts. The results suggest that the region significantly influenced the time to the first ANC visit. Women residing in the central region exhibited a 26% shorter time for their initial ANC visit compared to those living in the northern areas.

## Discussion

This research aimed to identify determinants contributing to women's early intention of antenatal care (ANC) services using cox proportional hazard model analysis, given the significant impact of early ANC visits on maternal and child health outcomes. Maternal and child health indicators in Bangladesh is a concern, with early and regular antenatal care (ANC) visits crucial for reducing maternal and neonatal mortality rates^15,25^. Despite efforts to improve awareness and accessibility, many women delay their first ANC visit, missing opportunities for timely interventions and health promotion during pregnancy^15,26^.

The World Health Organization (WHO) recommends women attend antenatal care (ANC) during the first trimester due to its crucial role during pregnancy^27^. However, adherence to this recommendation is low in many low- and middle-income countries, including Bangladesh. This study found that 40.4% of women participated in at least one ANC activity during the first trimester, higher than in several other countries^16,28^. However, this prevalence is lower than the worldwide average of approximately 58%^1^, and is also lower than findings from other studies conducted in various regions^29,30^.

The study findings reveal that women in the 25–29 age group are more likely to have an early intention for ANC visits compared to younger women, consistent with previous research. Tesfaye et al.^31^ suggesting that factors like increased awareness, maturity, and readiness to embrace maternal health responsibilities contribute to this trend among women in this age bracket. Women’s educational level positively impacts early ANC intention, with lower-educated women less likely to book their first ANC visit compared to those with higher education levels. Educated mothers tend to be more conscious and well-informed about the benefits of ANC, which is consistent with previous research findings^32^. Similarly, the educational level of the partner also shows a positive association with early ANC intention.

The study identified a positive association between wealth status and women's intention to seek early antenatal care (ANC). Furthermore, women in poor-income households were less likely to have a timely intention for their first ANC visit. This finding aligns with a previous study conducted by Ejeta et al.^29^, Manyeh et al.^33^. Mothers with a pregnancy intention are more likely to have an early intention for ANC visits compared to those without a specific pregnancy intention, supported by a meta-analysis conducted in Ethiopia^34^. The likelihood of having an intention for early antenatal care (ANC) increases with access to mass media, underscoring the role of mass media in raising awareness, as highlighted by Acharya et al.^35^. Lastly, the study reveals that women residing in urban areas are less likely to have an early intention for ANC compared to their rural counterparts, consistent with previous research findings^32,36^.

This study contributes significantly to the achievement of Sustainable Development Goals (SDGs) and aligns with the priorities of the Bangladesh Government in improving maternal and child health. By focusing on the survival analysis of early intention of antenatal care, the study addresses a critical aspect of reproductive healthcare. The findings provide valuable insights that can inform targeted interventions to enhance maternal healthcare services, ultimately contributing to SDG 3 (Good Health and Well-being) and SDG 5 (Gender Equality). Furthermore, the study sheds light on the factors influencing the timing of antenatal care attendance, offering actionable information for policymakers and healthcare practitioners. Understanding the determinants of early intention can guide the development of targeted health policies and interventions, aligning with Bangladesh’s commitment to achieving the SDGs by 2030.

## Strengths and limitations of the study

This study has some strong points. First, using nationally representative cross-sectional data means we get a good picture of the whole population. The way we pick people for the study is random and covers a large group, so we can make accurate general conclusions about Bangladesh and similar places. Second, the study reduces bias in picking people by looking at the entire population of a country and using a specific sampling method. Lastly, the DHS program, which we used, is transparent and has a high response rate. This helps us get accurate estimates when we analyze the data. Despite its strengths, this study also faced some limitations. Firstly, due to data constraints, the study couldn’t include several crucial factors that significantly contribute to women's early ANC intention. Secondly, the cross-sectional nature of the data allows us to draw conclusions about associations, but it doesn’t allow us to establish causal links.

## Conclusion

In conclusion, this study highlights the significant influence of various factors on the early intention to seek Antenatal Care (ANC). Women’s age, wealth, and regional standing emerge as crucial determinants of the desire for early ANC. Additionally, socioeconomic status, education, pregnancy intention, employment status, mass media access, and residence significantly impact the intention to receive early ANC. Notably, poor and middle-class women, those with unintended pregnancies, and rural women exhibit lower intentions for early ANC. The findings emphasize the importance of addressing these factors to promote timely and accessible ANC services, ultimately improving maternal and child health outcomes. For future research, efforts should be made to overcome data limitations, ensuring a more comprehensive understanding of the factors influencing early intention of antenatal care. Longitudinal studies could be considered to establish causal links between early antenatal care and improved health outcomes, providing a more nuanced understanding of the impact of timely healthcare access on maternal well-being. Overall, the study lays a foundation for continued research and policy initiatives aimed at advancing maternal health in Bangladesh.

## Data Availability

In this study, we used data from the 2017-18 BDHS which is available from (https://dhsprogram.com/data/).
